# Bis(μ-2-{bis­[(2-oxidobenzyl­idene)amino]­meth­yl}phenolato)bis­[(tetra­hydro­furan)­samarium(III)] tetra­hydro­furan disolvate

**DOI:** 10.1107/S1600536812015759

**Published:** 2012-04-18

**Authors:** Li Li, Yuan Zhou, Fugen Yuan

**Affiliations:** a School of Chemistry and Biochemistry, University of Science and Technology of Suzhou, Suzhou 215009, People’s Republic of China

## Abstract

In the centrosymmetric binuclear complex of the title solvate, [Sm_2_(C_21_H_15_N_2_O_3_)_2_(C_4_H_8_O)_2_]·2C_4_H_8_O, the Sm^III^ is coordin­ated in a distorted monocapped octa­hedral geometry by four O atoms and two N atoms from two tridentate deprotonated 2-{bis­[(2-oxidobenzyl­idene)amino]­meth­yl}phenolate ligands and an O atom of a tetra­hydro­furan (THF) mol­ecule. The Sm⋯Sm distance in the complex is 3.8057 (4) Å. Parts of the coordinating THF mol­ecule are disordered over two sets of sites in a 0.56 (3):0.44 (3) ratio. The complex and solvent mol­ecules are linked into a three-dimensional structure *via* C—H⋯O hydrogen-bonding inter­actions.

## Related literature

For general reports on the tripodal ligand 2-bis-(salicyl­idiene­­amino)­methyl­phenol, see: Nabulsi *et al.* (1988 ▶); Achim *et al.* (2001 ▶); Yu *et al.* (1991 ▶); Snyder *et al.*(1989 ▶); Chaudhuri *et al.* (1998 ▶); Illingsworth *et al.* (2002 ▶). For related structures, see: Howell *et al.* (1998 ▶); Liu *et al.* (1998 ▶); Dubé *et al.* (1998 ▶). For ionic radii, see: Shannon (1976 ▶).
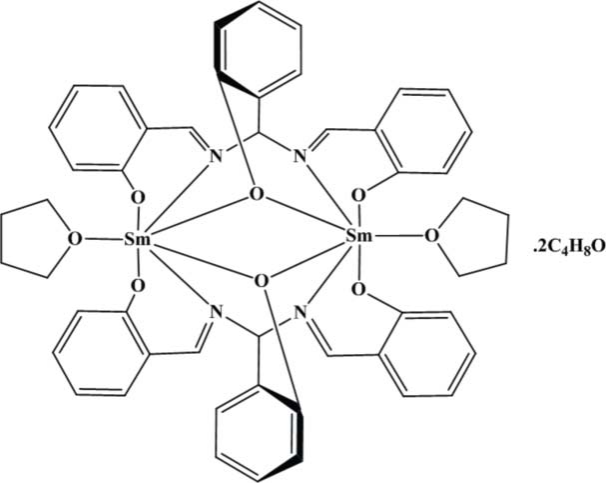


## Experimental

### 

#### Crystal data

[Sm_2_(C_21_H_15_N_2_O_3_)_2_(C_4_H_8_O)_2_]·2C_4_H_8_O
*M* *_r_* = 1275.84
Monoclinic, 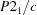

*a* = 11.7184 (11) Å
*b* = 21.378 (2) Å
*c* = 11.1464 (11) Å
β = 99.058 (1)°
*V* = 2757.5 (5) Å^3^
*Z* = 2
Mo *K*α radiation
μ = 2.17 mm^−1^
*T* = 293 K
0.23 × 0.18 × 0.16 mm

#### Data collection

Rigaku Rapid I CCD diffractometer
Absorption correction: multi-scan (*SADABS*; Bruker, 2007 ▶) *T*_min_ = 0.635, *T*_max_ = 0.723
23697 measured reflections
6291 independent reflections
5362 reflections with *I* > 2σ(*I*)
*R*_int_ = 0.052

#### Refinement

*R*[*F* ^2^ > 2σ(*F* ^2^)] = 0.032
*wR*(*F* ^2^) = 0.084
*S* = 1.01
6291 reflections
353 parameters
42 restraints
H-atom parameters constrained
Δρ_max_ = 0.62 e Å^−3^
Δρ_min_ = −0.49 e Å^−3^

### 

Data collection: *CrystalClear* (Rigaku, 2004 ▶); cell refinement: *CrystalClear*; data reduction: *CrystalStructure* (Rigaku/MSC, 2004 ▶); program(s) used to solve structure: *SHELXTL* (Sheldrick, 2008 ▶); program(s) used to refine structure: *SHELXTL*; molecular graphics: *SHELXTL*; software used to prepare material for publication: *publCIF* (Westrip, 2010 ▶) and *PLATON* (Spek, 2009 ▶).

## Supplementary Material

Crystal structure: contains datablock(s) I, global. DOI: 10.1107/S1600536812015759/wm2610sup1.cif

Structure factors: contains datablock(s) I. DOI: 10.1107/S1600536812015759/wm2610Isup2.hkl

Additional supplementary materials:  crystallographic information; 3D view; checkCIF report

## Figures and Tables

**Table 1:** Hydrogen-bond geometry (Å, °)

*D*—H⋯*A*	*D*—H	H⋯*A*	*D*⋯*A*	*D*—H⋯*A*
C4—H4*A*⋯O5	0.93	2.56	3.450 (7)	159
C21—H21*A*⋯O5^i^	0.93	2.56	3.424 (6)	155
C22—H22*B*⋯O3^ii^	0.97	2.50	3.079 (6)	118

Symmetry codes: (i) 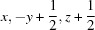; (ii) 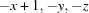.

**Fig. 1.**
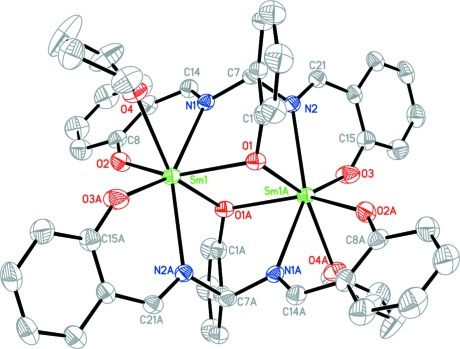
The molecular structure of the title compound with displacement ellipsoids scaled to 30% probability. H atoms and two uncoordinated THF molecules were omitted. The disorder of one of the THF molecules is not shown. [Symmetry code A: -x+1, -y, -z.]

**Fig. 2.**
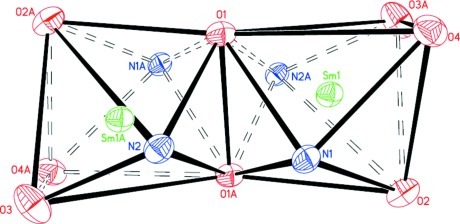
View of the distorted monocapped octahedral coordination geometry of SmIII with displacement ellipsoids scaled to 30% probability. [Symmetry code A: -x +1, -y, -z.]

**Fig. 3.**
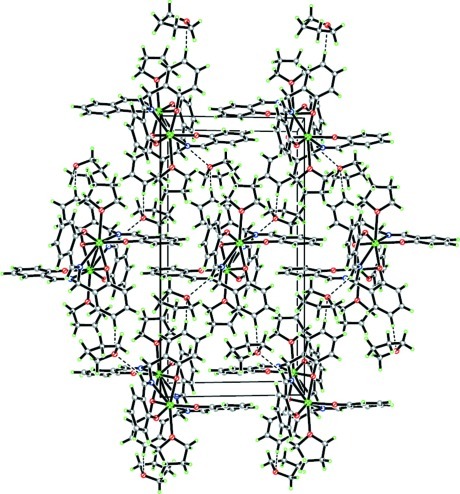
Packing diagram of the title compound viewed along [100]. Dashed lines indicate hydrogen bonds. The disorder is not shown.

## supplementary crystallographic information

### Comment

Since the tripodal schiff base 2-bis-(salicylidiene-amino)-methyl-phenol (salmp) has been characterized by Nabulsi (1988), it has been used as a chelating ligand for numerous transition metals, including Fe^II^, Fe^III^, Mn^II^, Mn^III^, Ti^III^, V^III^, Cr^III^, Co^III^ and Zr^IV^ (Achim *et al.*, 2001; Yu *et al.*, 1991; Snyder *et al.*, 1989; Chaudhuri *et al.*,1998; Illingsworth *et al.*, 2002). However, its coordination behaviour with respect to lanthanide elements has not yet reported to the best of our knowledge. Herein we reported a new dinuclear compound [Sm_2_(C_21_H_15_N_2_O_3_)_2_(C_4_H_8_O)_2_]^.^2(C_4_H_8_O), (I).

The molecular structure of compound (I) is presented in Fig. 1. The Sm—O bond lengths of 2. 213 (2) Å to the terminal aryloxide and of 2.369 (2) Å to the bridging aryloxide are similar compared to those in the analogous complex [{Sm(api)}_2_] (H_3_api = 2-(2-hydroxyphenyl)-1,3-bis[4-(2-hydroxyphenyl)-3-azabut-3-enyl]-1,3-imidazoline) (2.282 (4) and 2.388 (3) Å, respectively) (Howell *et al.*, 1998) when the difference of the ionic radii is taken into account. In the title compound, Sm has a coordination number of seven resulting in a distorted mono-capped octahedral coordination geometry for the metal (Fig. 2), whereas in the other structure the coordination number is eight. The radii of seven and eight-coordinate Sm^III^ are 1.160 and 1.219 Å (Shannon, 1976), respectively. The bond lengths of Sm—N (2.616 (2) to 2.623 (2) Å) are consistent with those in [(*η*^5^-(C_5_H_5_)Sm(*µ*-OC_20_H_20_N_2_O)]_2_.(*µ*-THF)^.^2THF (2.55 (5) to 2.67 (7) Å) (Liu *et al.*, 1998). The Sm···Sm distance in (I) is 3.8057 (4) Å, which is a little shorter than that in [(3,5-*Bu^t^*_4_salophen)Sm(OH)]_4_^.^4toluene (3.9023 (8) Å) (Dubé *et al.*, 1998). Two Sm atoms are bridged by aryloxide groups forming a Sm_2_(*µ*-O)_2_ planar rhomb with an acute angles O(1)—Sm(1)—O(1 A) of 73.11 (8)° and an obtuse angle Sm(1)—O(1)—Sm(1 A) of 106.89 (8)°.

The crystal packing is stabilized by C—H···O hydrogen bonds (Table 1, Fig. 3), including an intra-molecular hydrogen bond (C22—H22B···O3 ) and inter-molecular hydrogen bonds (C4—H4A···O5 and C21—H21A···O5).

### Experimental

2-bis-(salicylidiene-amino)-methyl-phenol was prepared according to the literature procedure (Illingsworth *et al.*, 2002). To a suspension of SmCl_3_ (0.536 g, 2.09 mmol) in 20 ml THF was added a THF solution of Ph_2_NK (10.3 ml, 6.27 mmol). The mixture was stirred for 1 d at room temperature. After centrifugation, a clear solution of Sm(NPh_2_)_3_ was obtained. To this solution was added the tripodal Schiff base (0.724 g, 2.09 mmol) and then stirred for another day. The resulting solution was concentrated and added with a proper volume of 1,2-dimethyloxyethane. Yellow crystals of [Sm_2_(C_25_H_23_N_2_O_4_)].2(C_4_H_8_O) were produced in a yield of *ca* 50%. m.p. > 250°C, IR(KBr, cm^-1^) 3391(w), 3043(w), 2974(w), 2866(w), 1610(*s*), 1541(*s*), 1468(*s*), 1445(*s*), 1406(*s*), 1316(*s*), 1267(*m*), 1189(w), 1148(*m*), 1123(w), 1026(w), 1011(*m*), 968(w), 914(w), 887(*m*), 852(w), 822(w), 749(*s*), 691(*m*), 578(w), 560(w), 452(w).

### Refinement

H atoms bound to tertiary C atoms were constrained to ideal geometry, with C—H = 0.98 Å and *U*_iso_(H) = 1.2*U*_eq_(C). The other H atoms were also placed in geometrically idealized positions and constrained to ride on their parent atoms, with C—H = 0.93 (aromatic and alkenyl) *U_iso_*(H) = 1.2*U*_eq_(C) or 0.97 Å (THF). The two central CH_2_- groups of the coordinating THF molecules were treated with disorder over two sites in a 0.56 (3) to 0.44 (3) ratio.

### Crystal data

**Table:**

[Sm_2_(C_21_H_15_N_2_O_3_)_2_(C_4_H_8_O)_2_]·2C_4_H_8_O	*F*(000) = 1284
*M**_r_* = 1275.84	*D*_x_ = 1.537 Mg m^−^^3^
Monoclinic, *P*2_1_/*c*	Mo *K*α radiation, λ = 0.71073 Å
Hall symbol: -P 2ybc	Cell parameters from 6291 reflections
*a* = 11.7184 (11) Å	θ = 1.8–27.5°
*b* = 21.378 (2) Å	µ = 2.17 mm^−^^1^
*c* = 11.1464 (11) Å	*T* = 293 K
β = 99.058 (1)°	Prism, yellow
*V* = 2757.5 (5) Å^3^	0.23 × 0.18 × 0.16 mm
*Z* = 2	

### Data collection

**Table:**

Rigaku Rapid I CCD diffractometer	6291 independent reflections
Radiation source: fine-focus sealed tube	5362 reflections with *I* > 2σ(*I*)
Graphite monochromator	*R*_int_ = 0.052
phi and ω scans	θ_max_ = 27.5°, θ_min_ = 1.8°
Absorption correction: multi-scan (*SADABS*; Bruker, 2007)	*h* = −15→15
*T*_min_ = 0.635, *T*_max_ = 0.723	*k* = −27→27
23697 measured reflections	*l* = −14→14

### Refinement

**Table:**

Refinement on *F*^2^	Primary atom site location: structure-invariant direct methods
Least-squares matrix: full	Secondary atom site location: difference Fourier map
*R*[*F*^2^ > 2σ(*F*^2^)] = 0.032	Hydrogen site location: inferred from neighbouring sites
*wR*(*F*^2^) = 0.084	H-atom parameters constrained
*S* = 1.01	*w* = 1/[σ^2^(*F*_o_^2^) + (0.0311*P*)^2^ + 2.4255*P*] where *P* = (*F*_o_^2^ + 2*F*_c_^2^)/3
6291 reflections	(Δ/σ)_max_ = 0.001
353 parameters	Δρ_max_ = 0.62 e Å^−^^3^
42 restraints	Δρ_min_ = −0.49 e Å^−^^3^

### Special details

**Table:**

Geometry. All e.s.d.'s (except the e.s.d. in the dihedral angle between two l.s. planes) are estimated using the full covariance matrix. The cell e.s.d.'s are taken into account individually in the estimation of e.s.d.'s in distances, angles and torsion angles; correlations between e.s.d.'s in cell parameters are only used when they are defined by crystal symmetry. An approximate (isotropic) treatment of cell e.s.d.'s is used for estimating e.s.d.'s involving l.s. planes.
Refinement. Refinement of *F*^2^ against ALL reflections. The weighted *R*-factor *wR* and goodness of fit *S* are based on *F*^2^, conventional *R*-factors *R* are based on *F*, with *F* set to zero for negative *F*^2^. The threshold expression of *F*^2^ > σ(*F*^2^) is used only for calculating *R*-factors(gt) *etc*. and is not relevant to the choice of reflections for refinement. *R*-factors based on *F*^2^ are statistically about twice as large as those based on *F*, and *R*- factors based on ALL data will be even larger.

### Fractional atomic coordinates and isotropic or equivalent isotropic displacement parameters (Å^2^)

**Table:**

	*x*	*y*	*z*	*U*_iso_*/*U*_eq_	Occ. (<1)
Sm1	0.633331 (14)	0.050642 (8)	0.045466 (14)	0.03765 (7)	
O1	0.4566 (2)	0.04480 (10)	−0.0885 (2)	0.0399 (5)	
N1	0.4689 (2)	0.08291 (12)	0.1669 (2)	0.0368 (6)	
C1	0.4042 (3)	0.10064 (16)	−0.1113 (3)	0.0415 (7)	
O2	0.7114 (2)	0.05416 (12)	0.2385 (2)	0.0510 (6)	
N2	0.2865 (2)	0.03847 (12)	0.0800 (2)	0.0376 (6)	
C2	0.4009 (4)	0.1298 (2)	−0.2238 (3)	0.0587 (10)	
H2B	0.4309	0.1096	−0.2858	0.070*	
O3	0.2239 (2)	−0.08830 (12)	0.0432 (3)	0.0640 (8)	
C3	0.3533 (4)	0.1885 (2)	−0.2430 (5)	0.0777 (14)	
H3A	0.3516	0.2075	−0.3182	0.093*	
C4	0.3082 (4)	0.2194 (2)	−0.1527 (5)	0.0732 (13)	
H4A	0.2778	0.2595	−0.1660	0.088*	
O5	0.1336 (5)	0.3502 (3)	−0.1746 (6)	0.1290 (16)	
C5	0.3088 (3)	0.19034 (17)	−0.0424 (4)	0.0570 (10)	
H5A	0.2766	0.2109	0.0178	0.068*	
C6	0.3561 (3)	0.13107 (16)	−0.0184 (3)	0.0425 (7)	
C7	0.3516 (3)	0.09823 (15)	0.0998 (3)	0.0401 (7)	
H7A	0.3123	0.1254	0.1511	0.048*	
C8	0.6870 (3)	0.05687 (15)	0.3492 (3)	0.0461 (8)	
C9	0.7750 (4)	0.0467 (2)	0.4482 (4)	0.0656 (12)	
H9A	0.8493	0.0377	0.4336	0.079*	
C10	0.7542 (5)	0.0496 (2)	0.5665 (4)	0.0794 (15)	
H10A	0.8145	0.0427	0.6301	0.095*	
C11	0.6450 (5)	0.0626 (2)	0.5916 (4)	0.0789 (15)	
H11A	0.6309	0.0646	0.6713	0.095*	
C12	0.5580 (4)	0.0726 (2)	0.4966 (3)	0.0645 (11)	
H12A	0.4843	0.0811	0.5133	0.077*	
C13	0.5755 (3)	0.07050 (17)	0.3742 (3)	0.0454 (8)	
C14	0.4763 (3)	0.08349 (16)	0.2826 (3)	0.0443 (8)	
H14A	0.4087	0.0937	0.3120	0.053*	
C15	0.1322 (3)	−0.07402 (19)	0.0914 (3)	0.0505 (8)	
C16	0.0517 (4)	−0.1210 (2)	0.1098 (4)	0.0672 (12)	
H16A	0.0643	−0.1620	0.0876	0.081*	
C17	−0.0452 (4)	−0.1066 (2)	0.1603 (4)	0.0672 (12)	
H17A	−0.0966	−0.1383	0.1721	0.081*	
C18	−0.0673 (3)	−0.0468 (2)	0.1935 (4)	0.0619 (11)	
H18A	−0.1333	−0.0377	0.2269	0.074*	
C19	0.0096 (3)	−0.0006 (2)	0.1764 (3)	0.0517 (9)	
H19A	−0.0054	0.0402	0.1984	0.062*	
C20	0.1104 (3)	−0.01278 (17)	0.1268 (3)	0.0425 (7)	
C21	0.1904 (3)	0.03821 (16)	0.1224 (3)	0.0419 (7)	
H21A	0.1698	0.0759	0.1546	0.050*	
O4	0.6278 (2)	0.17034 (12)	0.0626 (2)	0.0548 (7)	
C22	0.6431 (5)	0.2121 (2)	−0.0357 (4)	0.0754 (14)	
H22A	0.5697	0.2300	−0.0723	0.091*	
H22B	0.6767	0.1902	−0.0978	0.091*	
C23	0.7226 (16)	0.2615 (7)	0.0220 (11)	0.079 (4)	0.56 (3)
H23A	0.8018	0.2524	0.0127	0.094*	0.56 (3)
H23B	0.7014	0.3021	−0.0137	0.094*	0.56 (3)
C23'	0.647 (3)	0.2738 (6)	0.0115 (14)	0.088 (6)	0.44 (3)
H23C	0.5731	0.2945	−0.0144	0.106*	0.44 (3)
H23D	0.7064	0.2977	−0.0188	0.106*	0.44 (3)
C24	0.709 (3)	0.2603 (12)	0.146 (2)	0.138 (10)	0.56 (3)
H24A	0.6689	0.2977	0.1659	0.166*	0.56 (3)
H24B	0.7842	0.2595	0.1970	0.166*	0.56 (3)
C24'	0.671 (3)	0.2708 (12)	0.144 (3)	0.102 (8)	0.44 (3)
H24C	0.7510	0.2802	0.1741	0.122*	0.44 (3)
H24D	0.6220	0.2998	0.1803	0.122*	0.44 (3)
C25	0.6426 (6)	0.2047 (2)	0.1716 (5)	0.099 (2)	
H25A	0.6848	0.1804	0.2377	0.119*	
H25B	0.5685	0.2166	0.1930	0.119*	
C26	0.1419 (11)	0.3737 (5)	−0.0552 (11)	0.182 (4)	
H26A	0.1247	0.4181	−0.0561	0.219*	
H26B	0.2189	0.3671	−0.0104	0.219*	
C27	0.0526 (12)	0.3368 (7)	0.0020 (11)	0.210 (5)	
H27A	0.0870	0.3019	0.0501	0.252*	
H27B	0.0110	0.3632	0.0512	0.252*	
C28	−0.0164 (13)	0.3174 (7)	−0.1032 (16)	0.234 (7)	
H28A	−0.0423	0.2752	−0.0907	0.281*	
H28B	−0.0844	0.3440	−0.1166	0.281*	
C29	0.0337 (11)	0.3180 (5)	−0.2067 (13)	0.189 (5)	
H29A	0.0490	0.2758	−0.2318	0.227*	
H29B	−0.0159	0.3388	−0.2726	0.227*	

### Atomic displacement parameters (Å^2^)

**Table:**

	*U*^11^	*U*^22^	*U*^33^	*U*^12^	*U*^13^	*U*^23^
Sm1	0.04306 (11)	0.03983 (11)	0.03134 (10)	0.00188 (7)	0.00982 (7)	−0.00368 (6)
O1	0.0485 (13)	0.0387 (12)	0.0329 (12)	0.0053 (9)	0.0080 (10)	0.0013 (9)
N1	0.0417 (14)	0.0387 (14)	0.0301 (13)	0.0009 (11)	0.0057 (11)	−0.0049 (11)
C1	0.0417 (17)	0.0445 (18)	0.0359 (17)	−0.0001 (14)	−0.0015 (13)	0.0054 (14)
O2	0.0491 (14)	0.0656 (17)	0.0367 (13)	0.0100 (11)	0.0022 (11)	−0.0019 (11)
N2	0.0392 (14)	0.0422 (14)	0.0316 (14)	0.0018 (11)	0.0060 (11)	−0.0041 (11)
C2	0.069 (3)	0.063 (2)	0.042 (2)	0.002 (2)	0.0040 (18)	0.0136 (18)
O3	0.0641 (17)	0.0511 (15)	0.086 (2)	−0.0039 (13)	0.0419 (16)	−0.0080 (14)
C3	0.090 (3)	0.070 (3)	0.068 (3)	0.006 (3)	−0.005 (3)	0.032 (2)
C4	0.075 (3)	0.048 (2)	0.090 (4)	0.010 (2)	−0.005 (3)	0.022 (2)
O5	0.146 (4)	0.104 (3)	0.140 (4)	−0.003 (3)	0.031 (3)	0.027 (3)
C5	0.052 (2)	0.044 (2)	0.074 (3)	0.0074 (17)	0.0041 (19)	−0.0034 (18)
C6	0.0416 (18)	0.0374 (17)	0.0472 (19)	0.0013 (13)	0.0029 (14)	0.0009 (14)
C7	0.0438 (18)	0.0424 (17)	0.0342 (16)	0.0055 (14)	0.0067 (13)	−0.0074 (13)
C8	0.060 (2)	0.0399 (18)	0.0357 (18)	0.0019 (15)	−0.0008 (15)	−0.0005 (13)
C9	0.069 (3)	0.076 (3)	0.047 (2)	0.016 (2)	−0.007 (2)	0.0040 (19)
C10	0.100 (4)	0.092 (4)	0.038 (2)	0.015 (3)	−0.016 (2)	0.008 (2)
C11	0.109 (4)	0.094 (4)	0.031 (2)	0.002 (3)	0.004 (2)	0.007 (2)
C12	0.079 (3)	0.082 (3)	0.034 (2)	−0.002 (2)	0.0131 (19)	0.0017 (19)
C13	0.054 (2)	0.0492 (19)	0.0322 (17)	−0.0051 (16)	0.0049 (15)	0.0009 (14)
C14	0.0471 (19)	0.052 (2)	0.0350 (17)	−0.0029 (15)	0.0118 (14)	−0.0041 (15)
C15	0.048 (2)	0.059 (2)	0.046 (2)	−0.0026 (17)	0.0133 (16)	0.0009 (17)
C16	0.065 (3)	0.059 (2)	0.083 (3)	−0.012 (2)	0.028 (2)	−0.005 (2)
C17	0.047 (2)	0.084 (3)	0.072 (3)	−0.016 (2)	0.015 (2)	0.003 (2)
C18	0.038 (2)	0.098 (3)	0.051 (2)	−0.001 (2)	0.0095 (17)	0.006 (2)
C19	0.0440 (19)	0.071 (2)	0.0399 (19)	0.0075 (17)	0.0066 (15)	0.0024 (17)
C20	0.0377 (17)	0.061 (2)	0.0281 (16)	0.0022 (15)	0.0029 (13)	0.0010 (14)
C21	0.0444 (18)	0.0512 (19)	0.0305 (16)	0.0064 (14)	0.0067 (14)	−0.0026 (13)
O4	0.0740 (18)	0.0430 (14)	0.0503 (15)	−0.0026 (12)	0.0192 (13)	−0.0008 (11)
C22	0.117 (4)	0.054 (2)	0.057 (3)	−0.003 (3)	0.018 (3)	0.013 (2)
C23	0.083 (8)	0.075 (7)	0.081 (7)	−0.023 (6)	0.025 (7)	0.012 (5)
C23'	0.129 (17)	0.041 (6)	0.093 (9)	−0.019 (8)	0.010 (10)	0.004 (6)
C24	0.23 (2)	0.093 (15)	0.078 (11)	−0.082 (15)	−0.006 (13)	−0.015 (9)
C24'	0.20 (2)	0.040 (7)	0.082 (12)	−0.008 (10)	0.077 (14)	−0.004 (7)
C25	0.184 (7)	0.058 (3)	0.062 (3)	−0.022 (3)	0.038 (4)	−0.011 (2)
C26	0.216 (8)	0.150 (7)	0.170 (7)	−0.023 (6)	−0.001 (7)	−0.007 (6)
C27	0.209 (9)	0.256 (9)	0.175 (8)	0.040 (7)	0.066 (7)	0.020 (7)
C28	0.231 (10)	0.228 (10)	0.262 (10)	−0.026 (8)	0.096 (9)	−0.013 (8)
C29	0.175 (10)	0.126 (8)	0.259 (15)	0.000 (7)	0.013 (10)	0.013 (9)

### Geometric parameters (Å, º)

**Table:**

Sm1—O2	2.203 (2)	C15—C20	1.402 (5)
Sm1—O3^i^	2.223 (3)	C15—C16	1.414 (5)
Sm1—O1	2.358 (2)	C16—C17	1.380 (6)
Sm1—O1^i^	2.380 (2)	C16—H16A	0.9300
Sm1—O4	2.568 (3)	C17—C18	1.365 (6)
Sm1—N1	2.616 (3)	C17—H17A	0.9300
Sm1—N2^i^	2.623 (3)	C18—C19	1.371 (6)
Sm1—Sm1^i^	3.8057 (4)	C18—H18A	0.9300
O1—C1	1.348 (4)	C19—C20	1.405 (5)
O1—Sm1^i^	2.380 (2)	C19—H19A	0.9300
N1—C14	1.279 (4)	C20—C21	1.443 (5)
N1—C7	1.494 (4)	C21—H21A	0.9300
C1—C2	1.396 (5)	O4—C25	1.407 (5)
C1—C6	1.413 (5)	O4—C22	1.446 (5)
O2—C8	1.312 (4)	C22—C23'	1.419 (14)
N2—C21	1.289 (4)	C22—C23	1.486 (12)
N2—C7	1.487 (4)	C22—H22A	0.9700
N2—Sm1^i^	2.623 (3)	C22—H22B	0.9700
C2—C3	1.375 (6)	C23—C24	1.42 (3)
C2—H2B	0.9300	C23—H23A	0.9700
O3—C15	1.310 (4)	C23—H23B	0.9700
O3—Sm1^i^	2.223 (3)	C23'—C24'	1.46 (3)
C3—C4	1.379 (7)	C23'—H23C	0.9700
C3—H3A	0.9300	C23'—H23D	0.9700
C4—C5	1.376 (6)	C24—C25	1.47 (3)
C4—H4A	0.9300	C24—H24A	0.9700
O5—C29	1.357 (11)	C24—H24B	0.9700
O5—C26	1.412 (11)	C24'—C25	1.49 (3)
C5—C6	1.392 (5)	C24'—H24C	0.9700
C5—H5A	0.9300	C24'—H24D	0.9700
C6—C7	1.501 (5)	C25—H25A	0.9700
C7—H7A	0.9800	C25—H25B	0.9700
C8—C9	1.403 (5)	C26—C27	1.527 (14)
C8—C13	1.409 (5)	C26—H26A	0.9700
C9—C10	1.380 (7)	C26—H26B	0.9700
C9—H9A	0.9300	C27—C28	1.379 (16)
C10—C11	1.381 (8)	C27—H27A	0.9700
C10—H10A	0.9300	C27—H27B	0.9700
C11—C12	1.365 (7)	C28—C29	1.374 (15)
C11—H11A	0.9300	C28—H28A	0.9700
C12—C13	1.412 (5)	C28—H28B	0.9700
C12—H12A	0.9300	C29—H29A	0.9700
C13—C14	1.447 (5)	C29—H29B	0.9700
C14—H14A	0.9300		
			
O2—Sm1—O3^i^	101.30 (11)	C18—C17—C16	121.5 (4)
O2—Sm1—O1	143.97 (9)	C18—C17—H17A	119.2
O3^i^—Sm1—O1	112.64 (10)	C16—C17—H17A	119.2
O2—Sm1—O1^i^	87.80 (9)	C17—C18—C19	118.7 (4)
O3^i^—Sm1—O1^i^	142.00 (8)	C17—C18—H18A	120.7
O1—Sm1—O1^i^	73.11 (8)	C19—C18—H18A	120.7
O2—Sm1—O4	84.53 (9)	C18—C19—C20	122.2 (4)
O3^i^—Sm1—O4	72.66 (9)	C18—C19—H19A	118.9
O1—Sm1—O4	94.01 (8)	C20—C19—H19A	118.9
O1^i^—Sm1—O4	145.32 (8)	C15—C20—C19	119.0 (3)
O2—Sm1—N1	73.27 (9)	C15—C20—C21	123.2 (3)
O3^i^—Sm1—N1	143.28 (9)	C19—C20—C21	117.6 (3)
O1—Sm1—N1	72.33 (8)	N2—C21—C20	128.7 (3)
O1^i^—Sm1—N1	74.71 (8)	N2—C21—H21A	115.6
O4—Sm1—N1	70.69 (8)	C20—C21—H21A	115.6
O2—Sm1—N2^i^	114.64 (9)	C25—O4—C22	108.6 (3)
O3^i^—Sm1—N2^i^	70.63 (9)	C25—O4—Sm1	125.7 (3)
O1—Sm1—N2^i^	88.65 (8)	C22—O4—Sm1	123.5 (2)
O1^i^—Sm1—N2^i^	72.04 (8)	C23'—C22—O4	107.1 (7)
O4—Sm1—N2^i^	141.16 (8)	O4—C22—C23	104.8 (6)
N1—Sm1—N2^i^	145.30 (8)	C23'—C22—H22A	76.2
C1—O1—Sm1	113.65 (19)	O4—C22—H22A	110.8
C1—O1—Sm1^i^	126.4 (2)	C23—C22—H22A	110.8
Sm1—O1—Sm1^i^	106.89 (8)	C23'—C22—H22B	136.3
C14—N1—C7	114.4 (3)	O4—C22—H22B	110.8
C14—N1—Sm1	125.9 (2)	C23—C22—H22B	110.8
C7—N1—Sm1	119.56 (18)	H22A—C22—H22B	108.9
O1—C1—C2	120.8 (3)	C24—C23—C22	104.4 (11)
O1—C1—C6	119.5 (3)	C24—C23—H23A	110.9
C2—C1—C6	119.7 (3)	C22—C23—H23A	110.9
C8—O2—Sm1	143.3 (2)	C24—C23—H23B	110.9
C21—N2—C7	114.0 (3)	C22—C23—H23B	110.9
C21—N2—Sm1^i^	126.8 (2)	H23A—C23—H23B	108.9
C7—N2—Sm1^i^	119.02 (18)	C22—C23'—C24'	108.8 (13)
C3—C2—C1	120.0 (4)	C22—C23'—H23C	109.9
C3—C2—H2B	120.0	C24'—C23'—H23C	109.9
C1—C2—H2B	120.0	C22—C23'—H23D	109.9
C15—O3—Sm1^i^	145.1 (3)	C24'—C23'—H23D	109.9
C2—C3—C4	121.1 (4)	H23C—C23'—H23D	108.3
C2—C3—H3A	119.4	C23—C24—C25	110.3 (14)
C4—C3—H3A	119.4	C23—C24—H24A	109.6
C5—C4—C3	119.1 (4)	C25—C24—H24A	109.6
C5—C4—H4A	120.5	C23—C24—H24B	109.6
C3—C4—H4A	120.5	C25—C24—H24B	109.6
C29—O5—C26	110.9 (9)	H24A—C24—H24B	108.1
C4—C5—C6	122.0 (4)	C25—C24'—C23'	103.6 (18)
C4—C5—H5A	119.0	C25—C24'—H24C	111.0
C6—C5—H5A	119.0	C23'—C24'—H24C	111.0
C5—C6—C1	118.1 (3)	C25—C24'—H24D	111.0
C5—C6—C7	121.8 (3)	C23'—C24'—H24D	111.0
C1—C6—C7	120.1 (3)	H24C—C24'—H24D	109.0
N2—C7—N1	107.2 (2)	O4—C25—C24'	108.6 (11)
N2—C7—C6	111.1 (3)	O4—C25—C24	104.7 (10)
N1—C7—C6	112.7 (3)	O4—C25—H25A	110.8
N2—C7—H7A	108.6	C24'—C25—H25A	124.0
N1—C7—H7A	108.6	C24—C25—H25A	110.8
C6—C7—H7A	108.6	O4—C25—H25B	110.8
O2—C8—C9	119.4 (4)	C24'—C25—H25B	92.0
O2—C8—C13	122.8 (3)	C24—C25—H25B	110.8
C9—C8—C13	117.8 (4)	H25A—C25—H25B	108.9
C10—C9—C8	121.7 (5)	O5—C26—C27	105.1 (9)
C10—C9—H9A	119.1	O5—C26—H26A	110.7
C8—C9—H9A	119.1	C27—C26—H26A	110.7
C9—C10—C11	120.7 (4)	O5—C26—H26B	110.7
C9—C10—H10A	119.6	C27—C26—H26B	110.7
C11—C10—H10A	119.6	H26A—C26—H26B	108.8
C12—C11—C10	118.5 (4)	C28—C27—C26	98.6 (10)
C12—C11—H11A	120.7	C28—C27—H27A	112.1
C10—C11—H11A	120.7	C26—C27—H27A	112.1
C11—C12—C13	122.7 (4)	C28—C27—H27B	112.1
C11—C12—H12A	118.6	C26—C27—H27B	112.1
C13—C12—H12A	118.6	H27A—C27—H27B	109.7
C8—C13—C12	118.5 (4)	C29—C28—C27	116.1 (14)
C8—C13—C14	124.5 (3)	C29—C28—H28A	108.3
C12—C13—C14	117.0 (4)	C27—C28—H28A	108.3
N1—C14—C13	129.0 (3)	C29—C28—H28B	108.3
N1—C14—H14A	115.5	C27—C28—H28B	108.3
C13—C14—H14A	115.5	H28A—C28—H28B	107.4
O3—C15—C20	122.0 (3)	O5—C29—C28	104.4 (12)
O3—C15—C16	120.2 (4)	O5—C29—H29A	110.9
C20—C15—C16	117.8 (3)	C28—C29—H29A	110.9
C17—C16—C15	120.8 (4)	O5—C29—H29B	110.9
C17—C16—H16A	119.6	C28—C29—H29B	110.9
C15—C16—H16A	119.6	H29A—C29—H29B	108.9

Symmetry code: (i) −*x*+1, −*y*, −*z*.

### Hydrogen-bond geometry (Å, º)

**Table:**

*D*—H···*A*	*D*—H	H···*A*	*D*···*A*	*D*—H···*A*
C4—H4*A*···O5	0.93	2.56	3.450 (7)	159
C21—H21*A*···O5^ii^	0.93	2.56	3.424 (6)	155
C22—H22*B*···O3^i^	0.97	2.50	3.079 (6)	118

Symmetry codes: (i) −*x*+1, −*y*, −*z*; (ii) *x*, −*y*+1/2, *z*+1/2.
